# A true congenital pancreatic cyst in a dog

**DOI:** 10.1186/s12917-022-03215-6

**Published:** 2022-08-09

**Authors:** D. M. Healy, J. P. Cassidy, S. A. Martin

**Affiliations:** 1 Anderson Moores Veterinary Specialists, The Granary, Bunstead Barns, Poles Lane, Hursley, Winchester SO 21 2LL England; 2 From the section of Veterinary Clinical Sciences, School of Veterinary Medicine, University College Dublin, Belfield, Dublin 4 Ireland; 3 Veterinary Specialists Ireland, Clonmahon, Summerhill, Co., Meath, A83 EV27 Ireland

**Keywords:** Congenital pancreatic cyst, Canine, Histopathology

## Abstract

**Background:**

True congenital pancreatic cysts are a rare pathological process reported within feline and human literature. To date there has been no documented case of a true congenital cyst affecting a canine patient. The objective of this case report is to document the clinical findings, diagnostic investigations, surgical treatment, histopathological diagnosis and long-term outcome of a dog with a true pancreatic cyst.

**Case presentation:**

A 5-month-old crossbreed dog was presented with a six-week history of abdominal pain, apparent bilateral pelvic limb weakness, reluctance to walk and intermittent vomiting and diarrhoea. An abdominal ultrasound examination performed by the dog’s primary care veterinarian identified a large intra-abdominal structure of unclear origin. A computed tomographic examination identified a large ovoid structure measuring 156 mm in length, 95 mm in height and 89 mm in width and apparently originating from the left limb of the pancreas. An exploratory coeliotomy was performed and a partial pancreatectomy was performed to allow complete removal of the cystic structure. Histopathological analysis of sections of the wall of the large fluid-filled cyst identified a thick fibromuscular wall lined by a well regimented hyperplastic tall columnar epithelium with basally located round to ovoid nuclei featuring fine chromatin stippling and abundant apically located and surface mucin, concurrent with a true congenital pancreatic cyst. A long-term follow-up of twenty-nine months identified no clinical signs of recurrence.

**Conclusion:**

A partial pancreatectomy and en bloc excision of a true pancreatic cyst provided an excellent long-term outcome in a dog.

## Background

Cystic lesions of the pancreas are infrequently documented within both the veterinary and human literature. Isolated case reports and series identify pseudocysts [1, 2], cystic neoplastic lesions [3] and pancreatic dilations [4]. Reports of pancreatic cysts identified in children may be divided into six categories: congenital, retention, duplication, pseudocyst, neoplastic, and parasitic cysts [5]. However, by definition a cyst is a closed sac having a distinct membrane and developing abnormally in a body cavity or structure (Merriam-Webster 2021.); true pancreatic cysts are extremely rare and thought to be congenital in origin [6]; the case presented herein describes the diagnosis, treatment and outcome of a true pancreatic cyst thought to be congenital in nature. To date there have been no previous documentation of congenital pancreatic cysts in canines.

## Case presentation

A five-month-old entire female crossbreed dog weighing 14.6 kg (32.2 lb) was presented for further investigations of insidious onset of abdominal pain, apparent bilateral pelvic limb weakness, reluctance to walk and intermittent vomiting and diarrhoea for a duration of six weeks prior to referral. Initially, clinical signs were managed with symptomatic therapies by the dog’s primary veterinarian. Due to the persistence and progressive worsening of observed signs, the dog’s primary veterinarian performed abdominal radiography and an abdominal ultrasound examination which identified a large 60 mm × 80 mm ovoid structure in the mid abdominal region. The primary veterinarian suspected mild inflammation of the surrounding mesentery and the remainder of the examination was considered normal at this time. An ultrasound-guided fluid sample was obtained from the structure and cytological analysis identified a proteinaceous fluid with necrotic cellular material and mild, non-septic neutrophilic inflammation with moderate to marked accumulation of uniformly blue-black pigment.

The patient was bright, alert and responsive during the examination, and all clinical parameters were within normal reference ranges. A non-reducible, non-painful umbilical hernia was identified. She displayed no obvious signs of pain or discomfort on abdominal palpation. The previously described abdominal cystic structure was not palpable on abdominal palpation.

Hematology and biochemistry analysis were grossly unremarkable and identified mild elevations in urea 15 mmol/l (3.6–8.6 mmol/l), creatinine 256 mmol/l (20-120 mmol/l), alkaline phosphatase (ALP) 167 U/L (0-82 U/L), alanine aminotransferase (ALT) 83 U/L (0–36 U/L) and amylase 2642 U/L (400–1300 U/L). Urinalysis of a sample obtained by cystocentesis identified epithelial 1+, red blood cells 1+, white blood cells 4+, and was therefore considered normal.

**Fig. 1:**
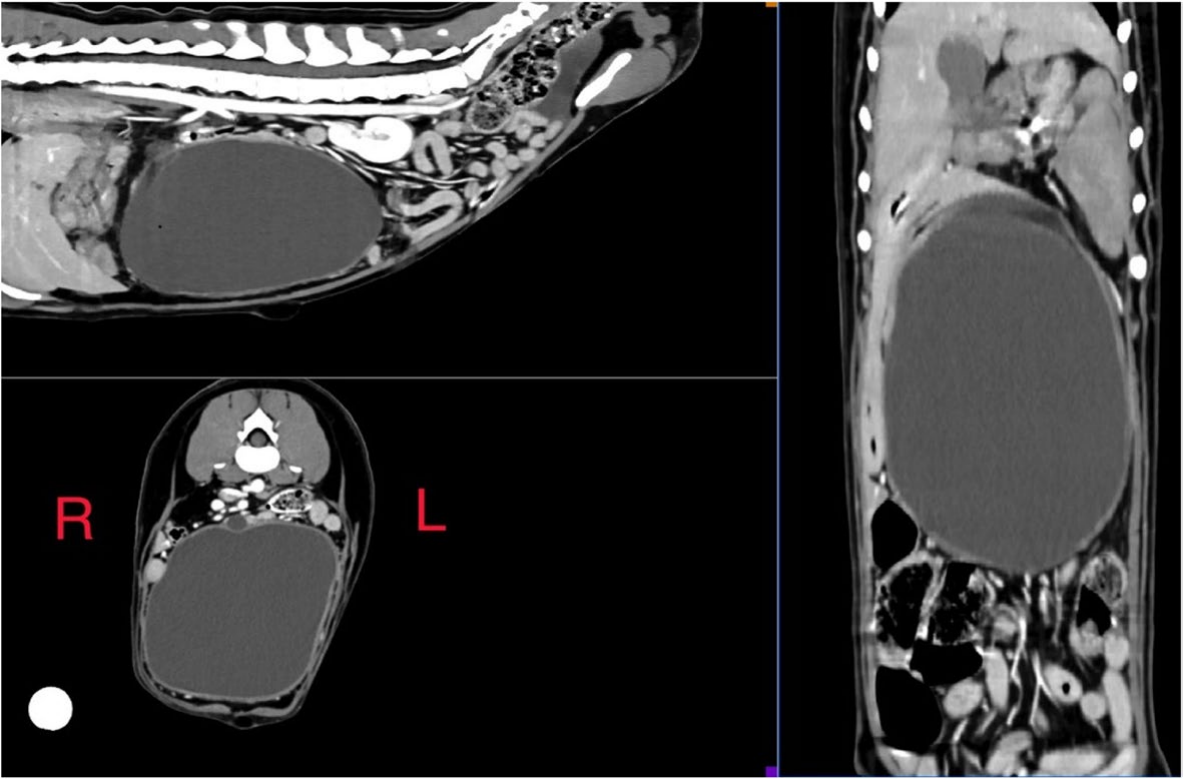
Multiplanar reconstruction of post-contrast abdominal computed tomography demonstrating the size, location, and discrete nature of the pancreatic cyst

A computed tomographic (CT) examination was performed under general anaesthesia. A combination of butorphanol 0.3 mg/kg (0.13 mg/lb) (torbugesic 10 mg/ml)^1^ and medetomidine hydrochloride 1μg/kg (0.0004 mg/lb) (sedastart 1 mg/ml,)^2^ were administered intravenously as a premedication. The patient was induced with Propofol 3 mg/kg (1.3 mg/lb) (propofol-lipuro 10 mg/ml)^3^ and a size 8 endotracheal tube was placed to allow maintenance of inhalant anaesthesia via sevoflurane in oxygen. Abdominal CT was performed with a 16-slice helical CT scanner (SOMATOM Scope; Siemens, Erlangen, Germany); pre- and post-intravenous contrast administration (ioversol 600 mg/kg (272 mg/lb) (optiray 300 mg/ml)).^4^ A large ovoid structure measuring 156 mm in length, 95 mm in height and 89 mm in width was identified, which was centrally fluid attenuating and non-contrast enhancing. It had a thin, approximately 3 mm thickness soft tissue and contrast enhancing margin, and was centered within the midventral abdomen. The mass extended from immediately caudal to the level of the gastric body (at the level of the T12 vertebral body and caudally to the level of the L3 vertebral body. At its maximum cross-sectional area (CSA) at the level of the L2-L3 intervertebral disc space, the mass occupied 75% the CSA of the abdominal cavity (Fig. 1). There was a focal thickening of the dorsal aspect of the wall of the mass measuring approximately 40 mm length, 47 mm height and 29 mm width, with one well defined ovoid (13 mm × 8.4 mm diameter) fluid attenuating structure located within the cranial aspect of this focally thickened tissue. A second focal nodular ovoid soft tissue attenuating and contract enhancing structure (9 mm × 6.3 mm) was located at the left ventrolateral margin of the large mass. The mass border effaced the ventral margin of the left limb of the pancreas. The mass displaced the left kidney caudally to the level of the L4-L6 vertebral bodies and displaced multiple segments of the small intestine caudally. The transverse colon was displaced dorsally. Multiple jejunal lymph nodes were moderately enlarged, up the 14 mm, but remained regularly marginated.

A sample of fluid was obtained from the structure which had a moderate cellularity in a moderately-basophilic, lightly and finely granular background with numerous red blood cells (RBCs) and acellular debris. Biochemical analysis of the fluid sampled identified a total protein level of less than 20 g/l, albumin less than 10 g/l, amylase and lipase values of 15,570 U/L and 1150 U/L were measured, respectively and a glucose concentration of 3.57 mmol/l.

On the day of surgical exploration, the dog was pre-medicated with medetomidine hydrochloride 1μg/kg (0.0004 mg/lb) (sedastart 1 mg/ml) and methadone 0.2 mg/kg (0.09 mg/lb) (synthadon 10 mg/ml).^5^ She was co-induced using a combination of ketamine 1 mg/kg (0.45 mg/lb) (ketamidor 100 mg /ml)^6^ and propofol 1 mg /kg (0.45 mg/lb) (propofol-lipuro 10 mg/ml). An appropriate plane of anaesthesia was maintained using sevoflourane in oxygen. As additional analgesia she was administered a morphine epidural at 0.2 mg/kg (0.9 mg/lb) diluted in 5 ml saline. A fentanyl (fentadon 50μg/ml)^7^ continuous rate infusion (CRI) at a rate of 1-2μg/kg/hr. (0.0004–0.0008 mg/lb./hr), following a loading dose of 2μg/kg (0.0008 mg/lb) IV and Lidocaine (lidocaine hydrochloride 5% solution)^8^ CRI at a rate of 1–3 mg/kg/hr. (0.45–1.3 mg/lb./hr) following a loading dose of 2 mg/kg (1.3 mg/lb) IV were also used as adjunctive analgesia based on her nociceptive response to surgery, as deemed appropriate by the attending anaesthetist.

**Fig. 2:**
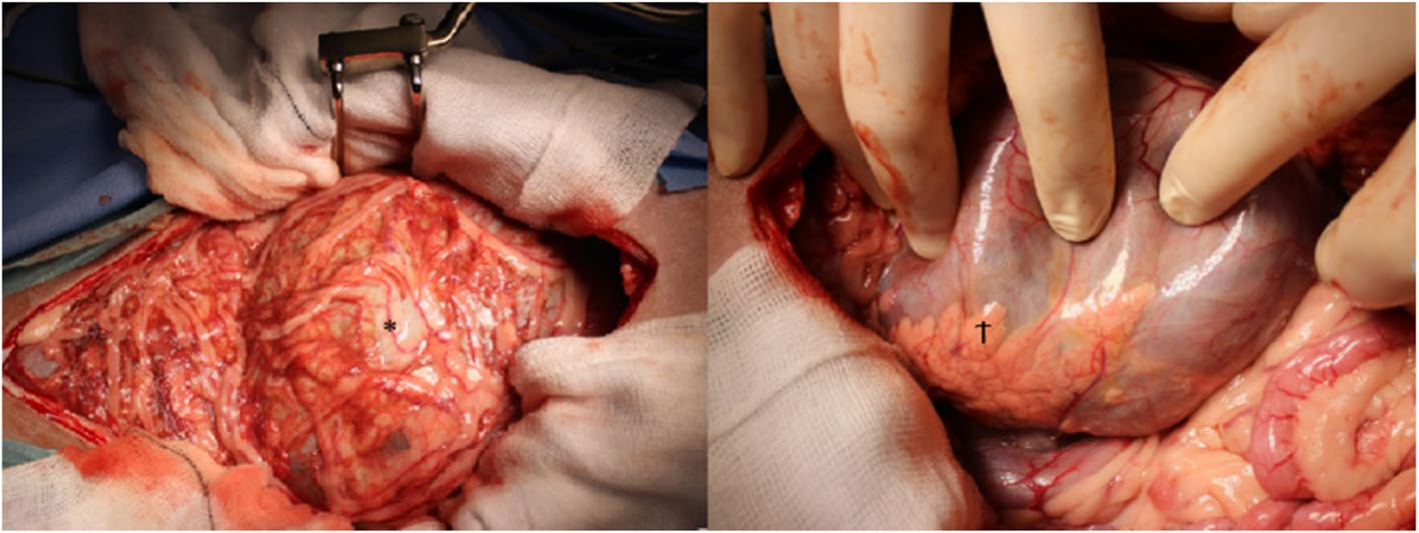
Intra operative: Left: The initial approach into the abdominal cavity identifying the gross omental overlay of the cyst (*). Right: The cystic structure and its association with the pancreas (†)

**Fig. 3:**
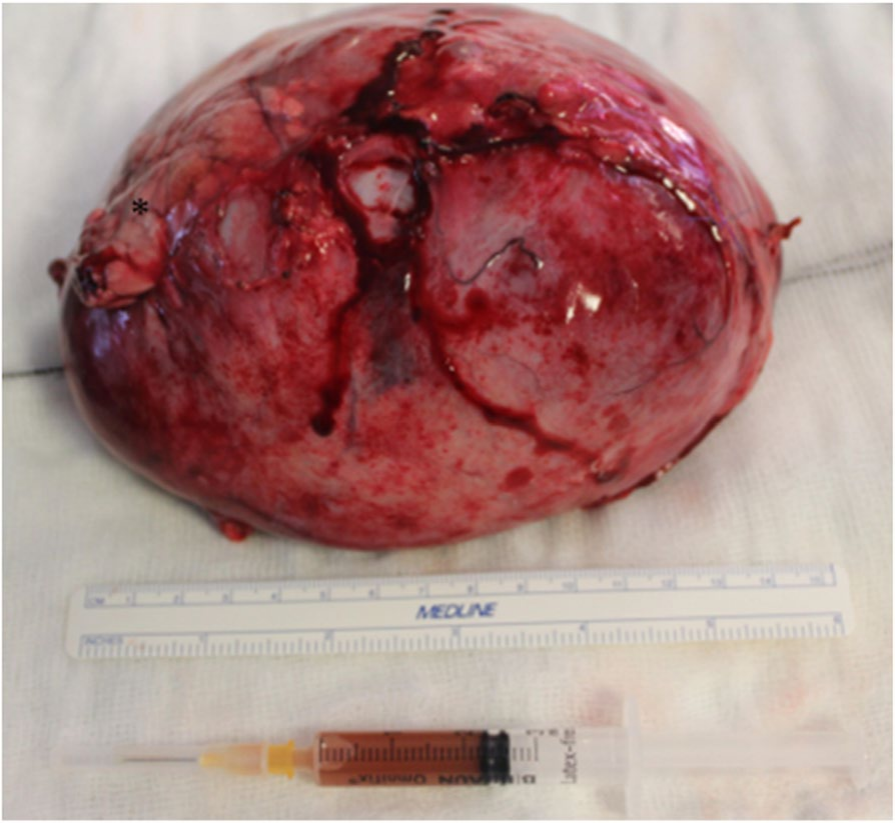
The cyst following the en-block excision, note the (*) highlighting the left limb of the pancreas and a syringe with a sample of the cystic contents

A standard ventral midline coeliotomy was performed from the xiphoid process to the pubis. The large ovoid structure was identified in the cranial mid-abdomen, consistent with the computed tomographic description of location and was confirmed to arise from the pancreas, at the junction between the left and the right limbs. The right limb of the pancreas was grossly normal and present within its physiological anatomical location. The left limb of the pancreas was oedematous and confluent with the surface of the mass. There was minimal adhesion of the mass to the remaining intra-abdominal structures. A partial pancreatectomy, the left limb was performed to allow complete removal of the cystic structure, en-bloc (Figs. 2 and 3).

Additional procedures performed, included ovariectomy, repair of the non-reducible umbilical hernia and the placement of an oesphagostomy feeding tube, due to risk of likely development of pancreatitis subsequent to surgery. No intra-operative complications were encountered. Abdominal closure was performed using 2.0 polydioxanone (2.0 PDS, Ethicon)^9^ for the external rectus sheath in a simple continuous pattern, 3.0 poliglycapone (3.0 Monocryl 3.0, Ethicon)^10^ for the subcutis and the 3.0 Nylon (3.0 Ethilon, Ethicon)^11^ for cutaneous cruciate sutures.

**Fig. 4:**
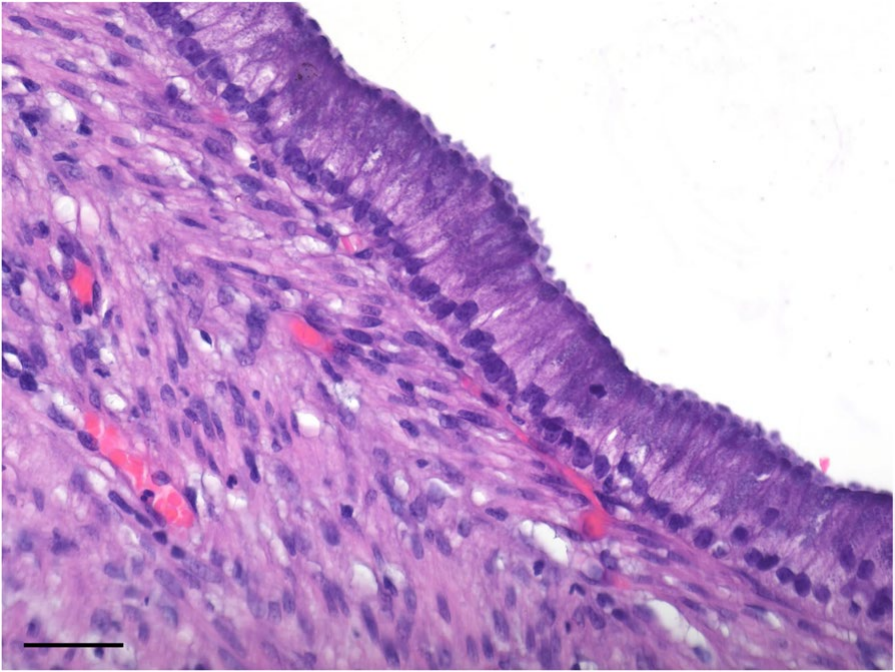
Histopathological examination of the cystic mass identified a thick fibromuscular wall lined by well regimented tall columnar epithelium (1 mitotic figure per 2 hpfs counted) with finely granular basophilic material in the apical cytoplasm and basally located round to ovoid nuclei featuring fine chromatin stippling

The entire mass was submitted to the institutional anatomic pathology department and sections of the wall (Fig. 3) of the mass were subsequently processed and sectioned for histopathological assessment. Histopathological assessment identified a thick fibromuscular wall lined by a well regimented hyperplastic tall columnar epithelium was confirmed with basally located round to ovoid nuclei featuring fine chromatin stippling and abundant apically located and surface mucin. A small focus of normal appearing pancreatic tissue was also noted intimately associated with the cyst wall in one submitted section (Fig. 4). This picture was captured using an Olympus EP50 microscope digital camera mounted on an Olympus BX50 microscope. The lesion was photographed at a magnification of × 400. The diagnosis of a true pancreatic cyst was confirmed and thought to be congenital in nature and derived from the larger tributaries of the pancreatic ductular system.

The dog recovered uneventfully from anaesthesia and was maintained in the hospital intensive care unit overnight. Within 12 h of surgery finish, the dog was eating and drinking voluntarily, and the oesophagostomy feeding tube was not employed. Post-operative analgesia was provided via a continuation of the fentanyl (fentadon 50μg/ml)^12^ and lidocaine (lidocaine hydrochloride 5% solution)^13^ CRI for the initial hours after surgery and once discontinued maintained on methadone 0.2 mg/kg (0.09 mg/lb) (synthadon 10 mg/ml)^14^ IV if deemed painful according to the Glasgow Pain scale [7], which in this case was only given once.

The dog was discharged from hospital three days post-operatively with orally administered non-steroidal anti-inflammatory medication, meloxicam 0.1 mg/kg (0.045 mg/lb) once daily (metacam 1.5 mg/ml).^15^ She also had instructions including exercise modification and wound care advice. A twenty-nine month follow-up was available for the patient via telephone conversation with the owners. Her owners reported no abnormalities, no clinical signs and described her as a normal dog.

## Discussion and conclusions

Congenital pancreatic cysts are a rare condition and infrequently reported in both the veterinary and human literature. A relatively recent article within the human literature identified only 25 reported cases of congenital pancreatic cysts [6] and as few as 4 case reports of true pancreatic cysts were identified within the veterinary literature; all in cats [8–11]. To the authors knowledge, at the time of writing, this is the first description of a congenital pancreatic cyst in dogs.

In human medicine, congenital pancreatic cysts are found preferentially in the neck or tail (62%) and the head (32%) of the pancreas or can diffusely involve the whole pancreas (6%) [12]. These growths are typically small in diameter (2–3 cm), but they may grow to great size in some cases. Congenital cysts may be solitary or multiple, and they may exist independently or in conjunction with a systemic disease such as von Hippel–Lindau syndrome or polycystic kidney disease. This association is more frequently found in patients with multiple congenital cysts, which can be so numerous they transform the pancreas into a cystic mass [13].

The exact aetiology of congenital pancreatic cysts remains uncertain at this time [5] but they are believed to be caused by a developmental anomaly of the pancreatic ductal system [14]. The failure of embryonic ducts to regress when they are replaced by permanent ducts during development may lead to their obstruction, creating a cyst that can fill with fluid. Congenital cysts are typically asymptomatic and are often found incidentally. However, in cases where the cyst is particularly large, or when it applies pressure to the surrounding viscera, it may cause abdominal distension, vomiting, jaundice or pancreatitis [6]. This was also evident in the case presented here which developed secondary clinical signs as a result of pressure on adjacent viscera and no biochemical abnormalities were identified. There are several case reports within human literature identifying infants with congenital cysts having various other congenital malformations, including Beckwith-Wiedemann syndrome [15], hemi hypertrophy [15]. A congenital comorbidity, albeit minor, was identified in our patient, having concurrent diagnosis of an umbilical hernia.

The current recommendation within the human literature regarding diagnostic investigation is computed tomography (CT) with iodinated contrast medium or magnetic resonance (MR) with gadolinium. Both diagnostic imaging modalities can achieve a diagnosis and help identify any evidence of malignant transformation, however, their accuracy in differentiating benign cysts from potentially malignant cysts is limited and varies between 20 and 80% depending on studies [16]. Fine needle aspiration (FNA) may help in the differential diagnosis of pancreatic cystic lesions (PCLs); however, the major challenge in the differential diagnosis of PCLs is to accurately discriminate between asymptomatic benign cysts and malignant ones (or those with malignant potential). It is essential to avoid under-diagnosis of pancreatic cystic lesions with malignant potential in order to allow a proper follow-up or curative surgical therapy [16]. Giving the rarity of both pancreatic cysts and pancreatic neoplasia this differentiation is of less importance within veterinary medicine and is difficult to postulate this importance without further cases.

Once a pancreatic cyst is identified, cystic fluid analysis can be useful to differentiate between the various types of pancreatic cystic lesions: congenital, retention, duplication, pseudocyst, neoplastic, and parasitic cysts [5]. Within the human literature, the concentration of pancreatic enzymes, lipase and amylase are utilised to differentiate the various types of cystic lesions. An amylase concentration of > 5000 U/ml had a 94% sensitivity and a 74% specificity for distinguishing pseudocysts from other cystic lesions in one publication [17]. This is also evident within the veterinary literature; documented in a feline patient with a pseudocyst and cystic amylase concentration > 5000 U/ml [18]. However, there are documented exceptions with a definite diagnosis of a congenital cyst having cystic amylase concentrations of < 5000 U/ml [19]. To the authors knowledge, to date this is the first documentation of the cystic fluid analysis of a definite diagnosis of a congenital pancreatic cyst of a canine patient. Therefore, drawing conclusions of its analysis has limited interpretation at this time and further investigations will be necessary to determine the efficacy of this analysis within the veterinary sphere. The use of canine pancreatic lipase immunoreactivity (cPLI) has been documented to have a high (90.9%) sensitivity for the diagnosis of acute pancreatitis as well as pancreatic neoplasms [20]. CPLI was not performed for this case. However, a pre- and post-operative cPLI could have been useful to monitor the development of post-operative pancreatitis, yet in this particular case there was no clinical indication of pancreatitis post-operatively. Additionally, the intra-cystic measurement of cPLI would be a further interesting diagnostic tool and further research in this area would be necessary.

The role of glucose concentration in intracystic fluid as a marker in the differential diagnosis of pancreatic cysts was first proposed by Park et al. in 2013. In particular, glucose concentration can be used to differentiate mucinous from non-mucinous cysts [19]; mucinous cysts have malignant potential whereas non-mucinous cysts do not [21] An intra-cystic glucose concentration of greater than 50 mg/dl (2.8 mmol/l) had sensitivity, specificity and accuracy of 96, 93.6 and 94.6% respectively for the identification of non-mucinous cysts [16]. The intra-cystic glucose concentration of this particular case was 3.57 mmol/l, correlating with previous literature,

A definitive diagnosis of a congenital pancreatic cyst can be obtained through histopathological analysis. True congenital pancreatic cysts are described to be lined with cuboidal-to-columnar epithelium [14] or simple cuboidal epithelium [22] with a deeper layer of acinar tissue. Histological examination of the structure described in this case identified a thick fibromuscular wall lined by well regimented columnar epithelium with basally located round to ovoid nuclei featuring fine chromatin stippling and abundant apically located and surface mucin.

While a complete cystectomy is the traditionally preferred treatment for a congenital pancreatic cyst [5] If the lesion is located at the head of the pancreas the cyst cannot be separated or removed from the pancreas then drainage of the mass via a cystoduodenostomy or a Roux-en-Y cystojejunostomy may be preferable [5] which has only been described within human literature. As in the case described here, the remainder of the pancreas was grossly considered normal intra-operatively; similar findings have been reported in human literature by Howard (1989), who described a rudimental inter-operative diagnosis of congenital cyst prior to histological examination based on the gross appearance of the pancreas, without the appearance of any inflammatory reaction [23]. The histological examination of this cyst identified normal appearing pancreatic tissue intimately associated with the cyst wall. Samples of the remaining pancreatic tissue was not obtained due to the possibility of iatrogenic pancreatitis, and it was grossly normal at the time of surgery. Yet histologic analysis may have been beneficial to identify underlying pathologies.

The rarity of documented cases of congenital pancreatic cysts within the veterinary literature makes it difficult to delineate similarities or inconsistencies when compared to human medicine. The case presented here describes a confirmed histopathologically diagnosed true congenital pancreatic cyst with elevated cystic amylase levels. Further investigations and case reports will be required to assess the relevance and commonality of these findings. However, complete surgical excision of the cyst was deemed curative for this single case, and long-term follow-up yielded no recurrence of clinical signs, no cyst regrowth, and no adverse clinical effects of partial pancreatectomy in a juvenile patient.

## Data Availability

All date generated or analysed during this study are included in this published article.

## Abbreviations

ALP: Alkaline phosphatase
ALT: Alanine Aminotransferase
cPLI: Canine Pancreatic Lipase Immunoreactivity
CRI: Continuous rate infusion
CSA: Cross-sectional area
FNA: Fine needle aspiration
PDS: Polydioxanone

## Footnotes

1 Torbugesic; Zoetis Ireland, Dublin, Ireland.

2 Sedastart; Animalcare Ltd., York, UK.

3 Propofol-Lipuro; B. Braun, Melsungen, Germany.

4 Optiray; Guerbet, Roissy, France.

5 Synthadon; Animalcare Ltd., York, UK.

6 Ketamidor; Chanelle Pharmaceuticals manufacturing Ltd., Galway, Ireland.

7 Fentadon; Dechra pharmaceuticals Ltd., UK.

8 Lidocaine Hydrochloride; B. Braum Medical Ltd., Dublin 12, Ireland.

9 PDS II; Ethicon, USA.

10 Monocryl; Ethicon, USA.

11 Ethilon; Ethicon, USA.

12 Fentadon; Dechra pharmaceuticals Ltd., UK.

13 Lidocaine Hydrochloride; B. Braum Medical Ltd., Dublin 12, Ireland.

14 Synthadon; Animalcare Ltd., York, UK.

15 Metacam: Boehringer Ingelheim Vetmedica GmbH, Ingelheim, Germany.
